# Cognitive modeling for understanding interactions between people and decision support tools in complex and uncertain environments: A study protocol

**DOI:** 10.1371/journal.pone.0290683

**Published:** 2023-10-05

**Authors:** Isaac Molina, Edmundo Molina-Perez, Fernanda Sobrino, Mario Tellez-Rojas, Luis Serra-Barragan, Alejandra Mitzi Castellón-Flores, Yessica Orozco, Adolfo de Unanue, Fatima Rojas-Iturria

**Affiliations:** 1 School of Government and Public Transformation, Tecnologico de Monterrey, Mexico City, México; 2 Faculty of Higher Studies Iztacala, National Autonomous University of Mexico, Mexico City, Mexico; 3 Instituto Politecnico Nacional, Centro Interdisciplario de Ciencias de Salud, Ciudad de Mexico, Mexico; PLoS ONE, UNITED STATES

## Abstract

**Background:**

Recent advances in Computational Intelligence Tools and the escalating need for decision-making in the face of complex and uncertain phenomena like pandemics, climate change, and geopolitics necessitate understanding the interaction between these tools and human behavior. It is crucial to efficiently utilize the decision-makers cognitive resources in addressing specific problems.

**Methods:**

The main goal of this present protocol is to describe the effect that CITs (Computational Intelligence Tools) have on decisions made during complex and uncertain situations. It is an exploratory study with a mixed methodology. Solomon’s group experiment design includes a narrative analysis of cognitive features such as integrative complexity (IC), cognitive flexibility (CF), and fluid intelligence (FI). Additionally, measures of neural activity (NA), physiological measures (PM), and eye-tracking data (ET) will be collected during the experimental session to examine the marginal impact of these processes on decision outcomes (DO) and their relation to CIT capabilities. To achieve this objective, 120 undergraduate and graduate students involved in decision-making will participate as subjects. The approximate duration of the study will be 2 years. Strict adherence to the relevant ethical considerations will be maintained during the performance of the experimental tasks.

**Discussion:**

The study will provide valuable information on CITs’ effect on decision-making under complex and uncertain contexts. This will help to better understand the link between technology and human behavior, which has important implications. CIT designers can use future results and at the same time, it will be possible to understand cognitive, behavioral, physiological processes, and even the subjective assessment of individuals when they use technological tools to solve a problem.

## Introduction

Despite considerable advances in the application of computational intelligence tools in decision-making processes, decision-makers struggle to keep up with complex and uncertain phenomena such as pandemics, climate change, and geopolitics. In recent years, the sharp rise in the availability of modern computational intelligence tools, such as automated data collection methods, satellite image processing, machine learning, artificial intelligence, and highly disaggregated simulation models, has attracted the attention of decision scientists. These tools present a potential opportunity to generate, collect, and analyze new data to enhance decision-makers’ abilities to respond to rapidly evolving decision environments.

From this perspective, computational intelligence tools (CITs) could be used as cognitive prostheses that expand the cognitive bandwidth of decision-makers, enabling them to more efficiently utilize their cognitive resources to address uncertainty and complexity in decision situations [1,2]. The concept of cognitive bandwidth, proposed by Mullainathan and Shafir [3], explores information saturation that decreases a person’s cognitive abilities and executive control. The authors provide empirical evidence of this concept through decreased performance in decision-making processes, psychometric tests, and even behavior patterns, such as the inability to follow a diet or adhere to treatment.

Recent progress in cognitive science and computational intelligence offers an opportunity to develop an integrated theoretical framework to study the interaction of CITs with human decision-makers in high-stakes decision contexts. For example, CITs can improve the decision-making process in crises providing decision-makers with new data, enabling quick estimation of likely consequences in a broader range of circumstances, and facilitating the identification of key decision trade-offs [4]. However, if CITs are implemented in a way that overwhelms users with excessive detail and complexity. In that case, it can lead to cognitive overload, confusion, mistrust, or deadlock, particularly in circumstances that require rapid action.

An empirical investigation using cognitive modeling, behavioral experimentation, and neuroscientific methods can be employed to describe the interaction between humans and CITs in decision-making processes. Behavioral experiments provide controlled environments where researchers can implement hypothetical scenarios (e.g., decision-making situations) that expose participants to challenges and problems resembling real-world crises. These experiments can test how different types of CITs are used to resolve such situations. Neuroscientific methods can then be utilized to collect data on participants’ characteristics, self-reported experiences, emotional and cognitive indicators, and biometric measures. These data can be combined to develop cognitive models that describe how participants’ characteristics and experiences interact with CIT properties to shape specific decision-making outcomes. In this investigation, the variables of cognitive abilities and executive control will be specified separately to provide greater precision regarding the components of cognitive bandwidth. These variables are described in the variables section of this document.

The fusion of behavioral experimentation and neuroscientific methods combine a diverse set of scientifically recognized paradigms that can be used to develop an integrated cognitive model of decision-making under uncertainty and complexity. For example, by monitoring participants’ eye movement behavior, patterns of retinal activation can be linked to patterns of CIT use and the outcomes of specific decisions. Eye movements provide insights into participants’ homeostatic balance and the underlying information that shapes decision situations. This can help understand how decision-makers process CIT results and stimuli to react in complex and uncertain environments [5,6]. Furthermore, combining these approaches could shed light on the neurological and behavioral processes through which CITs mitigate the impact of environmental stressors (e.g., time pressure, information overload, and risk) on individuals’ ability to construct a causal structure between different agents, perspectives, and data sources, and integrate them into a coherent overall decision judgment to resolve a crisis (integrative complexity) [7].

This interdisciplinary approach can contribute to: 1) objectively illustrating the belief and value models of decision-makers, 2) identifying the impact and mechanisms through which CITs influence individuals’ integrative complexity traits, 3) supporting CIT interventions in crises, and 4) contributing to the development of current decision sciences. Ultimately, this integrative approach may result in formal cognitive decision-making models under uncertainty and complexity, providing the scientific community with a deeper understanding of the mechanisms by which CITs and decision-makers interact in rapidly evolving learning environments.

## Method

### Research question

What is the marginal impact (i.e., the difference between performance in the and control condition) of the components of the TICs on people’s cognition and performance in complex and uncertain decision-making environments?

### Main objective

Describe the effect of CITs on decisions made during complex and uncertain situations.

#### Secondary objectives

Determine the relationship of the experimental parameters of the TICs with a positive impact of decision-makers’ ability to deal with a complex and uncertain environment.

Determine the combination of experimental parameters of the TICs that positively impact the level of integrative complexity of individuals when faced with an ambiguous, or deeply uncertain problem.

Determine the combination of experimental parameters of CITs that lead to mistrust, excessive technological dependence, model rejection or stagnation in complex and uncertain environments.

Evaluate the marginal impact of CIT components on people’s cognitive bandwidth in complex and uncertain decision-making environments.

Evaluate the CIT components’ marginal impact on the integrative complexity of people in complex and uncertain decision-making environments.

### Hypothesis

#### Null hypothesis

There is no effect of the CITs on the decisions that are made in complex and uncertain scenarios.

#### Alternate hypothesis

There is an effect of the CITs on the decisions made in complex and uncertain scenarios.

## Study design

### Study description

This phase 0 randomized controlled study that utilizes the Solomon cluster experimental design to assess the relationship between using CITs and decisions made during complex and uncertain situations. Potential populations for this study include undergraduate and graduate students, and decision-makers, given their close involvement in day-to-day decision-making processes. Participants will engage in decision-making strategies using a standardized interface, focusing on a classic decision-making problem known as Ellsberg’s paradox. No incentives or monetary resources will be at stake during the study. The participants’ psychophysiological responses will be recorded: blood pressure volume, respiration rate, skin conductance level, and EEG activity. Additionally, participants will complete standardized pencil-and-paper intelligence tests.

### Fundamentals of study design

CITs and ambiguity are defined and controlled variables.

### Fundamentals for the population of research subjects

Educational and professional settings are full of decisions that can be tied directly to an outcome. Undergraduate, graduate, and professional students are usually people of legal age who express their authorization for the study. In addition, this population may reveal valuable decision-making insights from their settings that increase the study’s external validity.

### Rationale for stratification factors

The participants in this study will consist of undergraduate or graduate students, and professionals from Tecnológico de Monterrey. All participants will be recruited through their teachers using a script and subsequently contacted via email to schedule an appointment in the study environment. The study topics will not be disclosed in class, and there will be no rewards or penalties for participation. All participants must volunteer and provide informed consent approved by the ethics committee of Tecnológico de Monterrey in Mexico.

One hundred and twenty (undergraduate and graduate students) will be divided into four research groups (RG). Each RG will comprise 30 individuals, with equal representation of 15 men and 15 women from various disciplinary backgrounds. The assignment of participants to each research group will adhere to the following experimental criteria:

RG 1: No computational system or ambiguity

RG 2: Unambiguous computer system

RG 3: Computational system with ambiguity

RG 4: Ambiguity without a computer system

Participants in the research groups will be carried out randomly while considering factors such as age, educational level, and sex to reduce intergroup variability. Each subject will belong to only one group and will participate in a single experimental session to avoid maturation and memory effects.

Regarding cognitive abilities, these factors will be analyzed post hoc to identify cognitive patterns associated with using CITs within the studied sample. Stratification based on cognitive conditions will not be conducted a priori, as the literature reports contradictory results between these conditions and the use of CITs and integrative complexity [8,9].

### Recruitment and inclusion/exclusion/suspension criteria

Following a maximum variation sampling logic; (maximum variation sampling involves selecting a diverse range of variations on the dimension of interest to accurately document unique or varied variations that identify common patterns throughout the sample [10]. This type of sampling is ideal for the present research, considering the theoretical gaps observed in the literature regarding the relationship between cognitive variables and the use of decision support tools.

The participants will be 120 subjects (undergraduate, graduate, and professional students) will be divided into four research groups (RG). Each RG will be composed of 30 individuals (15 men and 15 women with diverse disciplinary backgrounds) according to Solomon’s group experimental design. All participants will be recruited voluntarily and give their informed consent for the study, approved by the Ethics Committee of the Tecnológico de Monterrey in Mexico. The study only contemplates healthy subjects, so the term patient will be changed to that of participants.

### Inclusion criteria

The inclusion criteria for this study are intentionally general, given the exploratory nature of the research. The aim is to investigate the use of CITs without imposing many predefined criteria. Only exclusion criteria reported in the relevant literature, which are most closely related to potential variability in the data, will be considered.

Participants over 18 years of age.

Being an undergraduate or postgraduate student or exercising a professional activity.

Their professional or academic environment will not be directly associated with the specific context of the research for decision-making.

Participants who signed the informed consent

### Exclusion criteria

All eligibility and exclusion criteria will be inquired when confirming the appointment, and candidates will be assessed based on self-reporting to determine their suitability to participate in the experimental session. If a suppose a candidate does not meet the eligibility criteria. In that case, an explanation&explanation will be provided, and they will be invited to remain interested in the study’s results and maintain communication with the research team. The emphasis is on ensuring that individuals do not feel excluded for social reasons but rather for technical reasons related to the study.

Being left-handed.

This feature is an exclusion criterion because the literature on the use of EEG reports that the left hemisphere may favor some skills, i.e. the language skills of right-handed people may be 20% higher than left-handed people [11].

History of drug abuse.

Substance abuse has been associated with neurological disorders associated with atypical measures in the use of electroencephalographic recording measures, including cocaine, opiates, ecstasy, and cannabis [12]. For these reasons, the subjects will be asked if they have a history of illegal substance abuse and if their answer is affirmative, the subjects will be excluded from their participation.

Conditions of vulnerability.

All research involves risks, and defining vulnerability is complex and challenging. Consequently, in the present study, any participant will be excluded if their social conditions, which include experiencing different forms of poverty or facing displacement difficulties, could be negatively impacted by participating in an unpaid activity for an extended period. Additionally, pre-existing health conditions, such as a history of mental health illness, pregnancy, or any reported illness by the subject, will serve as exclusion criteria due to the increased risk associated with interventions for these individuals.

Be a student of one of the researchers.

To prevent reactance, social influence, or submission phenomena, individuals involved in a teacher-student relationship with research team members will be excluded from this study due to concerns regarding potential issues of subordination.

History of epileptic or convulsive episodes.

Although the case is rare, the use of EEG is contraindicated in patients with a history of epilepsy or seizures unless it is performed for diagnostic purposes.

### Suspension criteria

Discomfort during the session.

This criterion refers to any sensations of discomfort, pain, or displeasure reported by the research subject. It will be specified before initiating any registration or placement of equipment on the participant, and emphasis will be placed on notifying the research team at any time during the session if such sensations arise. In such a situation, the study will be suspended, and any potential human error in equipment placement or stimulus presentation will be reviewed. The participant will be informed of the situation and given the option to restart the session. If the participant chooses not to continue, the session will be concluded, and they will be allowed to leave the premises when they feel ready to do so.

Presence of auras.

According to Fernández Torre [13], "The epileptic aura is the part of a seizure that occurs before the loss of consciousness and is remembered. The surface electroencephalogram often fails to detect changes during an isolated aura. A feeling of fear is the most common affective symptom associated with epileptic discharges of mesial temporal origin. Somatosensory auras may include numbness, tingling, electrical sensations, or occasionally pain."

For the reasons above the participants will be informed to be aware of the presence of perceptible sensations of unidentified origin, as described by Fernández. If any of these sensations occur, the study will be immediately terminated, and the participant will be kept under observation by the research team for at least 60 minutes before leaving the facility.

Reporting of stress or headache.

Any feelings of stress, such as frustration, intense anger, or sweating, or a headache, will warrant suspending the study. The participant will remain at rest until they state that they feel calm and ready to leave the facilities.

### Study evaluations

Informed consent forms and selection record.

The informed consent form will be provided to the participant at the beginning of the first session. It will be emphasized that they have ample time to read it carefully. Once the participant indicates that they have finished reading, any questions or doubts will be addressed. A brief explanation of the study, its objectives, and research questions will be provided. Only when the participant fully approves and understands the procedures will their signature be requested.

All collected informed consent forms will be securely stored in a locked cabinet in the investigator’s office. The research team will maintain a participation record to document the details of each selected participant and to confirm their eligibility or rejection based on the exclusion criteria.

### Research methodology

The research team will design a decision-making stimulus situation based on standardized decision-making paradigms validated in the existing literature, such as Ellsberg’s paradox. The characteristics of this decision-making situation will include non-linear behavior, multi-criteria results, ambiguity, and uncertainty. The stimulus parameters and narrative will be designed through computational experimentation to identify viable and robust interventions to serve as a baseline for analysis.

The stimulus situation will always be defined by choice interfaces that do not threaten the participants and will not involve monetary resources or real economic gains. These interfaces will be developed based on previously standardized situations described in the available literature, with defined parameters of ambiguity and validated performance in computational interfaces, such as Ellsberg’s paradox.

The Ellsberg paradox is an experimental challenge where participants make decisions about a possible outcome based on the probability of an event occurring [14]. This paradox is frequently used in decision-making theory [15,16]and has modulations in terms of ambiguity (amount of information offered to the decision-maker) and uncertainty (random result).

The experimental task of the Ellsberg paradox involves making a series of bets (without involving economic resources, only predictions) on the random selection of a colored ball in a lottery. The balls are red, green, or blue, and participants receive information about the total number of balls in a container, which varies in each round. Three decision conditions are presented to the participants. In the first condition, complete information is provided for each betting option, allowing participants to estimate which ball is most likely to be chosen. In the second condition, ambiguity is introduced by combining the frequency information of two balls into a single category, making it impossible for participants to determine which ball is more frequent and thus has a higher probability of being chosen in the lottery. In the third condition, participants are provided with a decision support tool that presents information about the probability of selecting each ball, considering all possible container frequency combinations (see Appendix 1 for images of the interface).

The CIT will be an expert system based on artificial intelligence (SSEE), utilizing previous knowledge from the literature and the response options of the stimulus situation. Artificial intelligence will simulate the knowledge of an expert to solve a specific problem effectively. The resulting CIT will be unanimously validated by three expert judges in the use of computational tools in decision-making.

Participants will be presented with the interface, and only two research groups (RGs) will be asked to use the CIT to study the situation. Participants will have time to explore the decision-making situation and be guided through various phases of the interface. The decisions made by the participants will be entered into the CIT, which will provide feedback on the results of their decisions.

The experimental procedures will be divided into three steps, following the Solomon group experimental design. According to McCambridge et al. [17], Solomon’s 4-group design involves randomly assigning participants to four possibilities: (a) an experimental group with evaluations, (b) an experimental group without evaluations, (c) a control group with no assessments, and (d) a control group with no assessments. This design enhances internal validity by controlling for the effects of the baseline estimate and the interactions between the intervention and the baseline assessments.

Given that the present study examines the use of CITs in situations of ambiguity, the Solomon group design is an appropriate measure to observe the interaction between these variables within a single experimental design. The following groups will be established for the sample:

RG 1: No computational system or ambiguity

RG 2: Unambiguous computer system

RG 3: Computer system with ambiguity

RG 4: Ambiguity without the computer system

The inclusion of participants in the research groups will be carried out randomly, considering stratification and pairing factors based on their age, educational level, and sex to reduce intergroup variability. Each participant will belong to only one group and will participate in a single experimental session to avoid maturation and memory effects.

Regarding cognitive abilities, these factors will be analyzed post hoc to identify cognitive patterns associated with the use of CITs within the studied sample. Stratification based on cognitive conditions will not be conducted a priori, as the literature reports contradictory results regarding the relationship between cognitive abilities, using the CITs, and integrative complexity [8,9].

The procedure consists of the following steps:

STEP 1: Each participant will be scheduled for a visit to the decision theater of the Tecnológico de Monterrey, where they will be assigned to an experimental group and participate in a session on the stimuli used in the experiments. All groups will participate in an orientation session. Two groups will use the CIT during the stimulus, while the others will not have access to it. Eye tracking, physical activity, and EEG waveband activity data will be recorded in real-time throughout the session.

STEP 2: The social and cognitive characteristics of the participants will be assessed through surveys and standardized tests during a second visit to the decision theater of the Tecnológico de Monterrey. Participants will complete two psychometric tests measuring Verbal Intelligence (Terman-Merrill) and non-verbal intelligence (Beta 4).

STEP 3: All groups will be scheduled for a follow-up session in the laboratory. During this session, participants will receive feedback on the study’s results, discuss their experience solving the stimulus, and reflect on the using of CITs. The final results will also be shared with the participants. If participants cannot attend this step, an individual dossier containing their results and variable information will be sent to them.

### Variables

Detailed technical specifications, including the specific instruments for measuring each variable, as well as the level and type of measurement, can be found in the variable table of this document (Table 1).

**Table 1:** Variable table with detailed technical specification.

VARIABLE	CONCEPT DEFINITION	OPERATIONA L DEFINITION	VARIABLE TYPE	MEASUR EMENT SCALE	VARIABLE VALUE
**Eye Tracking** **(ET)**	Eye tracking involves a series of cameras and infrared or near-infrared light sources that track the gaze behavior of one eye (monocular) or both eyes (binoculars) [18].	Values obtained from Tobii Prolab software after registration Equipment: Tobii Pro Glasses 3.0	Quantitative	Interval	Numerical
**Neural Activity** **(NA)**	The pattern of neural activity involves a network of millions of neurons spread over a wide area. Activity patterns for different types of brain function overlap, based on the characteristics they share [19].	Voltage at locations F3, Fz, F4, C3, Cz, C4, P3, POz and P4. The electrode positions were organized in the following areas: frontal (F) left (F3) right (F4) center (Fz), centrotemporal (CT) left (C3) right (C4) center (Cz), parietotemporal (PT) left (P3) right (P4), parietooccipital center (PO) (POz). The EEG signal represents a wide range of frequencies that are commonly divided into different frequency bands, such as alpha band: 8–13 Hz, beta band: 13–30 Hz. Equipment: EEG Flexiful MCA Brainquick Micromed, nine electrodes will be placed according to the 10–20 system	Quantitative	Interval	Numerical
**Psychophysiological measures (PM)**	Psychophysiological measures provide a discrete and implicit way to determine the affective or cognitive state of the user on the basis of mind-body relationships. In reality, they are physical signals from the human body that are generated in response to psychological changes and are measured by special equipment in real time [20].	BVP, RR and SC levels obtained through the experimental conditions. Equipment. Decoder: PROCOMP THOUGHT TECHNOLOG Y Sensors: THOUGHT TECHNOLOGY Skin conductance sensor THOUGHT TECHNOLOGY respiratory sensor: TTH-SA931 THOUGHT TECHNOLOGY HR/BVP Sensor: THOUGHT TECHNOLOGY	Quantitative	Interval	Numerical
**Cognitive flexibility (CF)**	Cognitive flexibility is a critical executive function that can be broadly defined as the ability to adapt behaviors in response to changes in the environment[21].	Terman-Merrill Intelligence Scale. Equipment: Terman-Merrill test	Quantitative	Interval	Numerical
**Fluid intelligence (FI)**	Fluid intelligence (Gf) is defined as the ability to reason and the ability to generate, transform and manipulate different types of novel information in real time [22].	General score on the BETA 4 intelligence scale. Equipment: BETA 4 Test	Quantitative	Interval	Numerical
**Integrative Complexity (IC)**	Integrative complexity is a measure of the intellectual style used by individuals or groups in information processing, problem solving, and decision making[23].	CI scoring via AUTOIC software and manual coding of respondents. Equipment: Standard Dell Laptop	Quantitative	Interval	Numerical
**Decision** **Outcomes (DO)**	Fundamental properties of decisions that predict the performance and outcome of decisions in the real world [24].	We will compare the decisions of the GRs in the stimulus by comparing how their decisions behave against the set of robust options that we identified a priori, during the development of the stimulus. This difference in performance will be recorded across a wide range of outcome attributes, e.g. cost, positive impact, negative impact, damage avoided, and others. Equipment: Standard Dell Laptop	Quantitative	Interval	Numerical
**CIT Capacities (CCIT)**	Practical adaptation and self-organizing concepts, paradigms, algorithms, and implementations that enable or facilitate appropriate actions (intelligent behavior) in complex and changing environments [25].	CIT automation degrees; specifically: non automation, static scanning of system properties, unique "what if" simulation capabilities, scenario analysis, optimization, exploratory modeling, vulnerability scanning, and automatic identification of strong alternatives. Equipment: Standard Dell Laptop	Discreet	Interval	Alphanumeric

Eye Tracking (ET)

Eye tracking involves a series of cameras and infrared light sources that track the gaze behavior of one (monoc

ular) or both eyes (binocular) [18]. Using the CIT during a decision-making simulation introduces perceptual and cognitive demands on the participants, asked with making intervention decisions. Using a CIT to support decision-making involves a heterogeneous interaction between visual perception and multiple cognitive processes, including memory retrieval, problem-solving, and decision-making.

During the experiments, the following eye-tracking parameters will be collected, as recommended by Duerrschmid & Danner et al. [26]:

Time to first fixation (TTFF): The time elapsed between the appearance of the CIT and the user fixating their gaze for the first time on an area of interest, measured in seconds.

First Fixation Duration (FFD): The duration a user fixates on their first fixation point.

Fixation Duration (FD): The duration of a fixation, measured in seconds.

Fixation Count (FC): The number of fixations on a product.

Dwell Duration (DD): The time elapsed between the user’s first fixation on a product and the next fixation off the product, measured in seconds.

Dwell Count (DC): The number of "visits" to an area of interest (AOI).

These measurements offer new opportunities to gain insights into the interpretive process of participants during the decision-making situation, including differences between the research groups (RGs), the heuristics and biases involved in visual perception and decision-making, and the underlying mechanisms, such as search and recognition errors [27].

All measurements will be conducted using Tobii Pro Glasses 3.0 equipment, and the research team will receive proper training from the product suppliers.

Neural Activity (NA)

Neural activity will be recorded from nine scalp locations: F3, Fz, F4, C3, Cz, C4, P3, POz, and P4 using the Flexiful MCA Brainquick Micromed EEG. The nine electrodes will be placed according to the 10–20 system. The electrode positions are organized as follows: frontal (F) left (F3), right (F4), center (Fz); centrotemporal (CT) left (C3), right (C4), center (Cz); parietotemporal (PT) left (P3), right (P4); and parietooccipital center (PO) (POz). The EEG signal represents a wide range of frequencies commonly divided into different frequency bands, such as the alpha band (8–13 Hz) and the beta band (13–30 Hz).

The electroencephalographic recording will be performed by team members with trained experience in retrieving electroencephalographic records as part of their curriculum.

The electroencephalographic recordings will be conducted simultaneously with the biofeedback devices and the eye-tracking glasses, ensuring no cable crossing. The recordings will take place in a spacious environment (approximately 50m2) to minimize interference from other electronic devices.

Psychophysiological measures (PM)

Psychophysiological measures offer a discrete and implicit means to determine the affective or cognitive state of the user based on mind-body relationships. Essentially, they are physical signals the human body generates in response to to psychological changes and are measured in real-time using specialized equipment [20]. In this specific study, we will focus on three types of psychophysiological measures: Blood Pressure Volume (BVP), Respiration Rate (RR), and Skin Conductance (SC).

Executive Control (EC)

The Terman-Merrill Intelligence Scale will be administered to assess the executive control and cognitive flexibility of the participants. This intelligence scale is derived from the Binet scale and has been adapted and standardized for the Mexican population. The scale provides two overall scores: a) general cognitive intelligence (CI), and b) learning capacity. Additionally, it provides individual scores for ten cognitive characteristics of the respondent:

- Information
- Comprehension
- Verbal meanings
- Logical selection
- Arithmetic
- Practical judgment
- Analogies
- Sentence ordering
- Classification
- Seriation

These scores will help to assess the participants’ cognitive abilities related to executive control and cognitive flexibility.

Fluid intelligence (FI)

The fluid intelligence of participants, which refers to their ability to think logically, analyze, and solve novel problems independently of prior knowledge, will be measured using the Beta 4 Intelligence scale.

This is a non-verbal intelligence scale that consists of five subscales designed to assess the respondent’s ability to perform various tasks:

- Decryption keys
- Non-logical images
- Similarities
- Completion
- Progression

These subscales provide valuable insights into the participant’s cognitive abilities and capacity to respond to different types of tasks, thereby assessing their fluid intelligence.

Integrative Complexity (IC)

The research team will evaluate existing frameworks for measuring integrative complexity, such as Conway et al.’s [28] automated framework and develop a procedure to measure the level of integrative complexity exhibited by participants when they engage in problem-solving tasks using laboratory-tested decision-making tools. To achieve this, we will record the experimental sessions and the participants’ response and utilize this data to automatically generate written transcripts of the sessions (our laboratory already possesses this capability). These transcripts will then be processed using word and phrase searches to enable the assessment of integrative complexity in individuals based on Conway’s AUTOIC tool.

Decision Outcomes (DO)

We will compare the decisions made by the research groups (RGs) in response to the stimulus by evaluating how their decisions align with the set of robust options identified a priori during the development of the stimulus. This comparison will involve assessing differences in performance across a wide range of outcome attributes, including but not limited to cost, positive impact, negative impact, damage avoided, and others.

The data relevant to the decisions will be analyzed by the research team as well as an external expert whose work focuses on the development of computational methods for studying social-ecological resilience under uncertainty and complexity, particularly in the context of developing simulation models for analyzing policy decisions.

CIT Capacities (CCIT)

The CIT supporting the stimulus situation will exhibit varying degrees of automation. The degrees of automation will be categorized as follows: no automation, static scanning of system properties, unique what-if simulation capabilities, scenario analysis, optimization, exploratory modeling, vulnerability analysis, and automatic identification of strong alternatives.

The research team will specifically design the tool for the decision-making task using an expert system based on artificial intelligence (SSEE) framework. These types of systems utilize programming to simulate the knowledge of an expert through artificial intelligence, enabling users to solve specific problems effectively.

### Statistical analysis techniques

#### Methods and models of data analysis according to the type of variables

We will utilize a cognitive model to integrate various data obtained from the study. Cognitive models describe how different aspects or variables interact and contribute to the observed behavior in empirical studies. Various influences come into play during the decision-making process, resulting in specific behaviors. Cognitive models provide valuable insights into the interconnected cognitive processes that contribute to the observed behavioral outcomes [5]. In this study, we propose to develop the following model:

$$DO_{i}=\overset{N}{\underset{j=1}{\sum }}\overset{P}{\underset{i=1}{\sum }}\overset{T}{\underset{k}{\sum }}f_{i}(f_{j}(ET),f_{j}(NA),f_{j}(EC),f_{j}(FI),CIT_{k})+\varepsilon$$

Therefore, a set of models will be trained using experimental data to describe the outcomes of individual decisions (i.e. *DO_i_*). Each outcome will be modeled as a combination of the aggregate characteristics of the research groups participating in the study (i.e., N) and their interactions with different versions of CIT supporting the war game (i.e., T), using a set of math functions. i.e., P) to describe these interactions (i.e., P).

**Fig 1:**
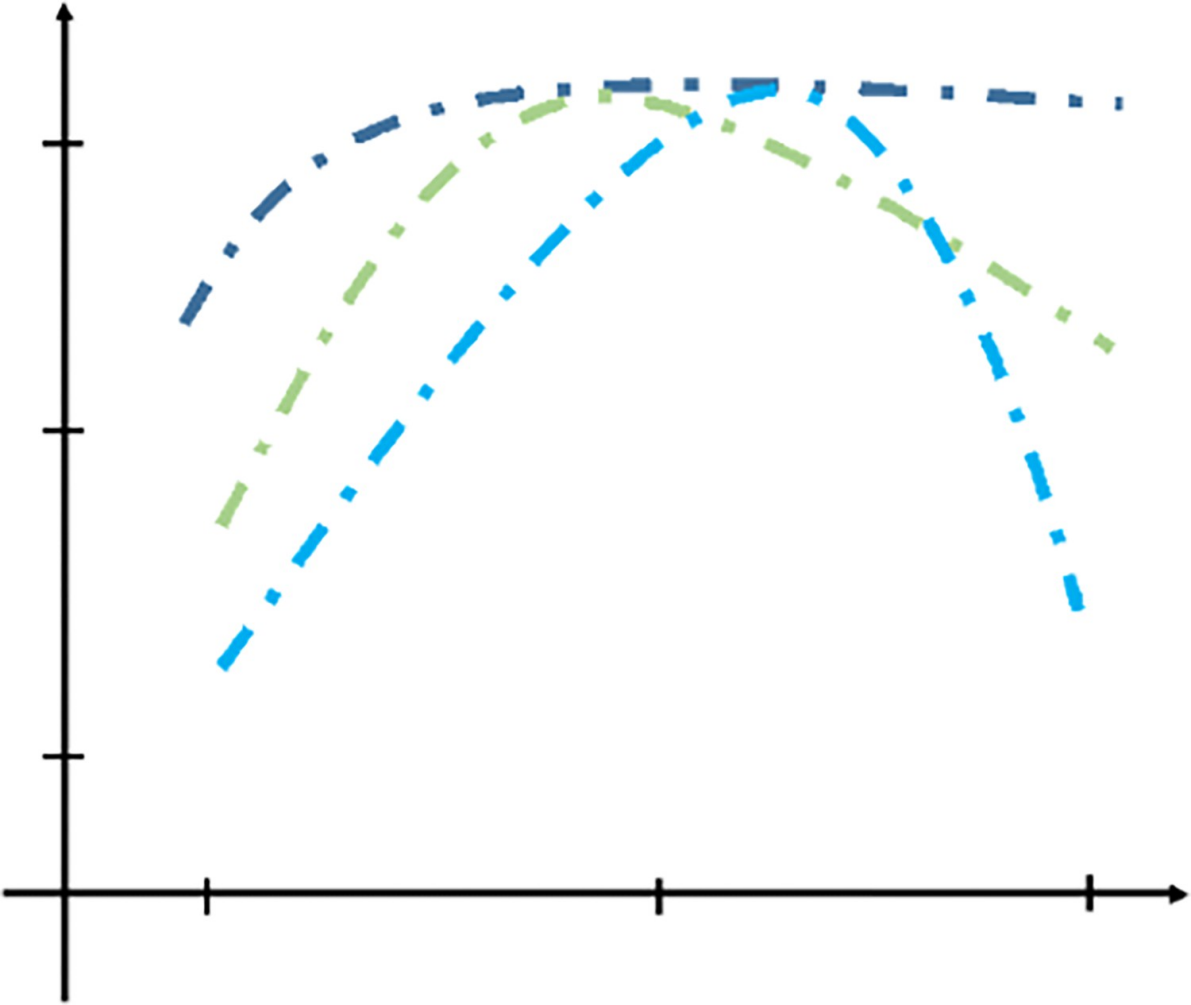
Automation and effectiveness tradeoffs for different cases.

Fig 1 presents a schematic example illustrating the expected outcomes of this research agenda. The scenario depicted in this figure suggests that the advantages of CITs peak at different levels of automation, depending on the specific planning team contexts. For instance, a group that with a substantial amount of pre-existing information may derive maximum benefits from CITs with moderate automation, as the abundance of available data may be associated with higher cognitive abilities. Conversely, a group with limited access to data may benefit from higher levels of automation to compensate for the lack of existing information. However, if the tools become overly complex for this group, the advantages of automation may diminish.

#### Programs to use for data analysis

Various software will be utilized based on the nature of the data extraction and analysis variables employed in this study.

Specifically:

Eye Tracking (ET)

Tobii ProLab v4.1 will be used to retrieve data from the eye-tracking instrument and for the analysis of variable metrics within the raw data.

Neural Activity (NA)

The Brainquick program will be employed to record and capture different signals from the EEG.

The Neuroguide program will be utilized to analyze the various numerical values of the EEG once extracted from the Brainquick software.

Psychophysiological measures (PM)

The Biograph v6 program will be used to analyze the specific sensor signals (BVP, RR, and SC) derived from the experimental situation and transform them into raw data.

Integrative complexity (IC)

The AUTOIC program takes verbal inputs, processes them, and provides a score for integrative complexity and its differentiation and integration components on a scale of 1–7.

Statistical programs

Once all the raw data is obtained, it will be computed and stored in a database. Python will be used for data processing, and statistical analysis of the data frames will be performed using RStudio software.

Principio del formulario

Regenerate responseFinal del formulario

### Ethical considerations

#### Ethics statement

This protocol has been approved by Instituto Tecnologico de Monterrey, Health Science Division ethics and scientific committee with invoice number P000737-CM- DMDU-CCT-CEIC-CR003 stating that this protocol in compliance with the guidelines of the GCP-ICH and local laws in force in Mexico; being present 6 members of the Ethics Committee on Research; the protocol, procedures and materials have been reviewed in methodological, technical and ethical aspects by the Ethics Committee of the Instituto Tecnológico y de Estudios Superiores de Monterrey, so that it is in the character of: Approved with validity of Approval Monday, March 06, 2023 to Tuesday, March 05, 2024. All participants must sign informed consent for their involvement in the study.

#### Compliance with laws and regulations

This study will be conducted in fully comply with the ICH E6 guideline of Good Clinical Practices and the principles of the Declaration of Helsinki and with the laws and regulations of Mexico or another country where the research is conducted, whichever provides the greatest protection to the individual.

#### Informed consent

A sample informed consent form from the sponsor will be provided at all centers. If applicable, it will be delivered in a certified local language translation. The sponsor or designated representative will review and approve any proposed deviations from the sponsor’s sample informed consent forms or any alternative consent forms proposed by the study site (collectively referred to as "consent forms") prior to submission to the internal review board and ethics committee (IRB/EC). IRB/EC approved consent forms will be provided to the sponsor for submission to health authorities following local requirements.

The informed consent form contains separate sections for optional procedures. The investigator or authorized designee will explain to each patient the goals, methods, and potential risks associated with each optional procedure. Patients will be informed that they are free to refuse participation and may withdraw their consent at anytime for any reason. A specific signature will be required to document a participant’s agreement to participate in elective procedures. Patients who decline to participate will not provide a separate signature.

The participant or legally authorized representative will sign and date the consent forms before participation in the study. Each patient’s case history or clinical records should document the informed consent process, and that written informed consent was obtained before participation.

Participants will re-consent with the most recent version of the consent forms (or with an addendum of significant new information/findings in accordance with applicable laws and IRB/EC policies) during their participation in the study. In the case of modified or updated consent forms, each patient’s case history or clinical records will document that the informed consent process and written informed consent were obtained using the updated/modified consent forms for continued participation in the study.

The participant will receive a copy of each signed consent form. All signed and dated consent forms must remain in each participant’s study file or site file and be available for verification by study monitors at any time.

#### Confidentiality

Once the informed consent is signed; all participant’s responses will be coded under a pseudonym. The only document with the participant’s real name will be the initial consent form, and all other data will remain encrypted with access restricted by password. Research involving direct patient contact.

The principal investigator maintains confidentiality standards by assigning a code to each participant included in the study using a unique patient identification number. This means that participant names are not included in the transmitted data sets.

The data obtained in this study is confidential and may only be disclosed to third parties as permitted by the informed consent form (or separate authorization to use and disclose personal health information) signed by the participant unless otherwise permitted or required by law.

Data generated in this study should be made available for inspection upon request by representatives of national and local health authorities and the IRB/EC, as appropriate.

#### Foreseeable and probable risks

None of the equipment provided in the study carries significant biohazards. Participants may experience mild stress of prolonged fasting due to the length of the experimental session.

#### Protection against physical and/or emotional risks

A certified psychologist will monitor and address any reported participant discomfort. If a participant reports stress, the psychologist may apply diaphragmatic breathing or other relaxation techniques as deemed appropriate. Additionally, medical services will be available in the study scenario to address participant emergencies. Diet services may be provided for sessions lasting more than 90 minutes.Principio del formulario Final del formulario.

## Discussion

The advancement of different scientific disciplines allows us to learn about important aspects of human behavior. Similarly, technologies have evolved, making it necessary to explore, understand, describe, and explain the strong link that has historically existed between the development of civilizations and decision-making [29].

To achieve this, we simultaneously consider the complexity and number of resources decision-makers require and the need to integrate a comprehensive model that includes cognitive, behavioral, and physiological aspects. Initially, we intend to analyze the data set separately but later integrate it to obtain a reliable description that captures the process and relationship between humans and machines (CITs). The decision-making process, whether in contexts of uncertainty and complexity, dynamic environments, confusing alternatives, or high-stress levels, involves various cognitive, emotional, and motivational processes that impose limitations on rational decision-makers [30]. A diverse range of data collection and measurement techniques will facilitate functional integration, enabling the description of the decision-making process and the relationship with CITs. Additionally, we plan to incorporate participants’ experiences into the analyses, providing insights into their beliefs, feelings, prejudices, and attitudes related to the conscious use of CITs.

Through this study, we aim to gain a better understanding of attentional, behavioral, and physiological processes, as well as to explore whether CITs can help mitigate the negative impact of factors such as stress (including time pressure, information overload, and potential risk) on decision-making for problem resolution [7]. Initially, we consider the Ellsberg paradox; however, we recognize the implications of social validity and acknowledge that people may not respond in the same way in a real-life situation as they would in a harmless experimental task. Therefore, in future task designs, we intend to adapt hypothetical situations that are more closely aligned with people’s reality, such as common health issues, personal finances, and experiences of violence. Janis and Mann [31] highlight that decision-making can lead to conflict and negative emotions when individuals face losses, whether subjective or not. This cognitive discomfort can affect self-evaluation and the estimation of desires, resulting in a negative sense of competition that influences future decisions. It is important to note that although decision-making appears to be individual, it is not strictly so, as it can impact other individuals, families, communities, cultures, or society. For example, the personal decision of whether to wear a face mask during the recent Covid-19 pandemic affected a group, and the consequences were significant.

### Study limitations

The authors recognize the need for a transdisciplinary approach and the use of mixed methodology to achieve the study objective, which may result in certain data sets being incomparable due to their inherent nature.

While the practicality of obtaining the sample focuses on participants of higher education levels, it is important to note that the decision-making process is not limited to professionals, business leaders, or individuals in public office. Decision-making is present from an early age and in daily life, such as deciding whether to wear a face mask, consume sugary foods, eat vegetables, or vote for a candidate. Therefore, future research, aims to include heterogeneous populations with hypothetical tasks beyond zero-risk bets, as real-life potential and actual losses have significant consequences.

In conclusion, this study will provide valuable information on the effect of CITs on decision-making in complex and uncertain contexts. This understanding will help shed light on the relationship between technology and human behavior, which has important implications for addressing global issues such as pandemics, economics, health, and geopolitics. The results may be utilized by CIT designers, may utilize the results while enhancing our understanding of individuals’ cognitive, behavioral, physiological processes, and subjective assessments of individuals when using technological tools to solve problems.

## Supporting information

S1 Fig Initial and final screens of experimental task. (TIF)

S2 Fig Phase 1 screens “Risk” (Game 1). (TIF)

S3 Fig Phase 2 screens “ambiguity” (Game 2). (TIF)

S4 Fig Phase 3 Screens “CIT” (Game 3). (TIF)

S5 Fig Feedback screens (positive and negative). (TIF)

S6 Fig Integrative Complexity questions screens. (TIF)
