# Supplementary figures and images for: Cognitive modeling for understanding interactions between people and decision support tools in complex and uncertain environments: A study protocol
Source: PLoS One. 2023 Oct 5;18(10):e0290683. doi: 10.1371/journal.pone.0290683 (PMC10553798; doi:10.1371/journal.pone.0290683)

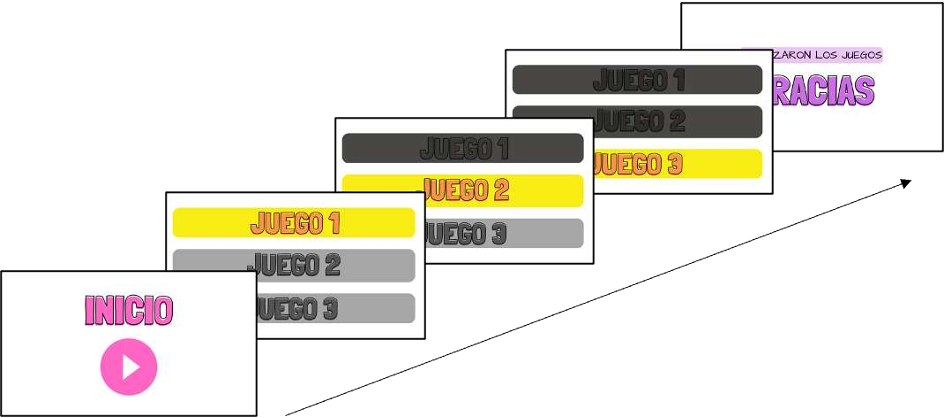

Supplement: S1 Fig — (TIF) [file pone.0290683.s001.tif]

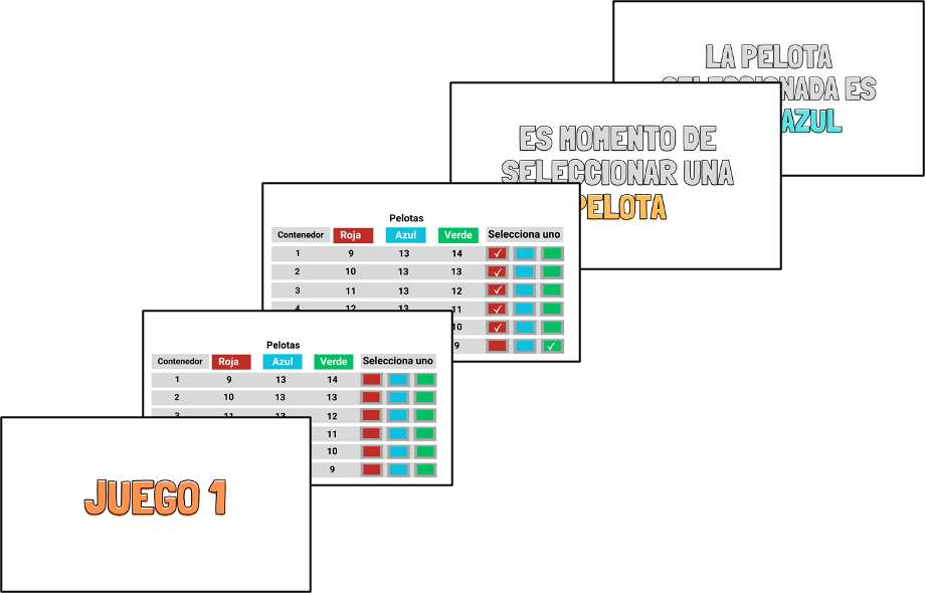

Supplement: S2 Fig — (TIF) [file pone.0290683.s002.tif]

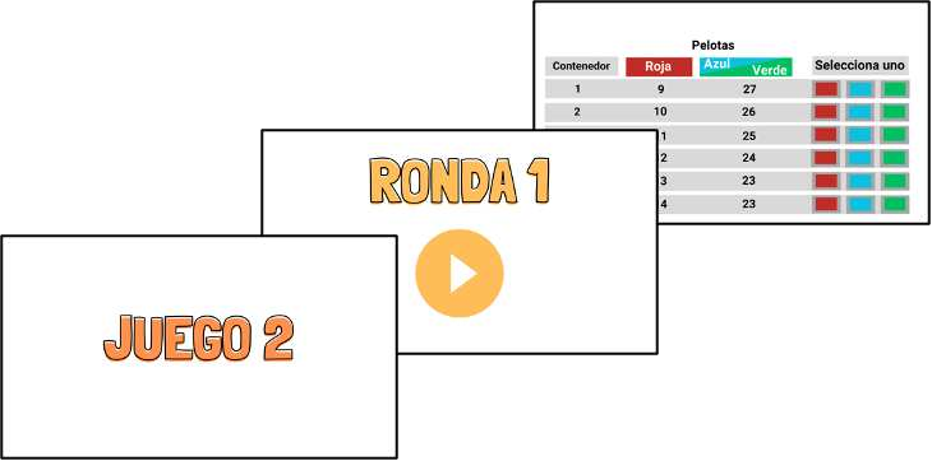

Supplement: S3 Fig — (TIF) [file pone.0290683.s003.tif]

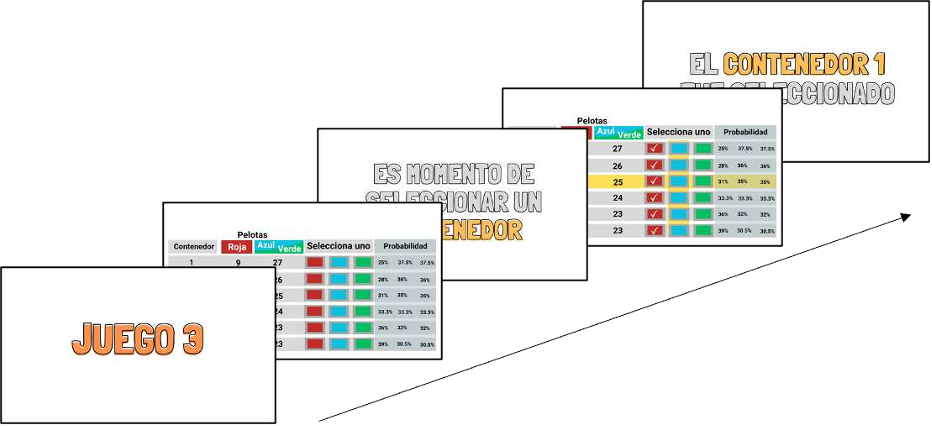

Supplement: S4 Fig — (TIF) [file pone.0290683.s004.tif]

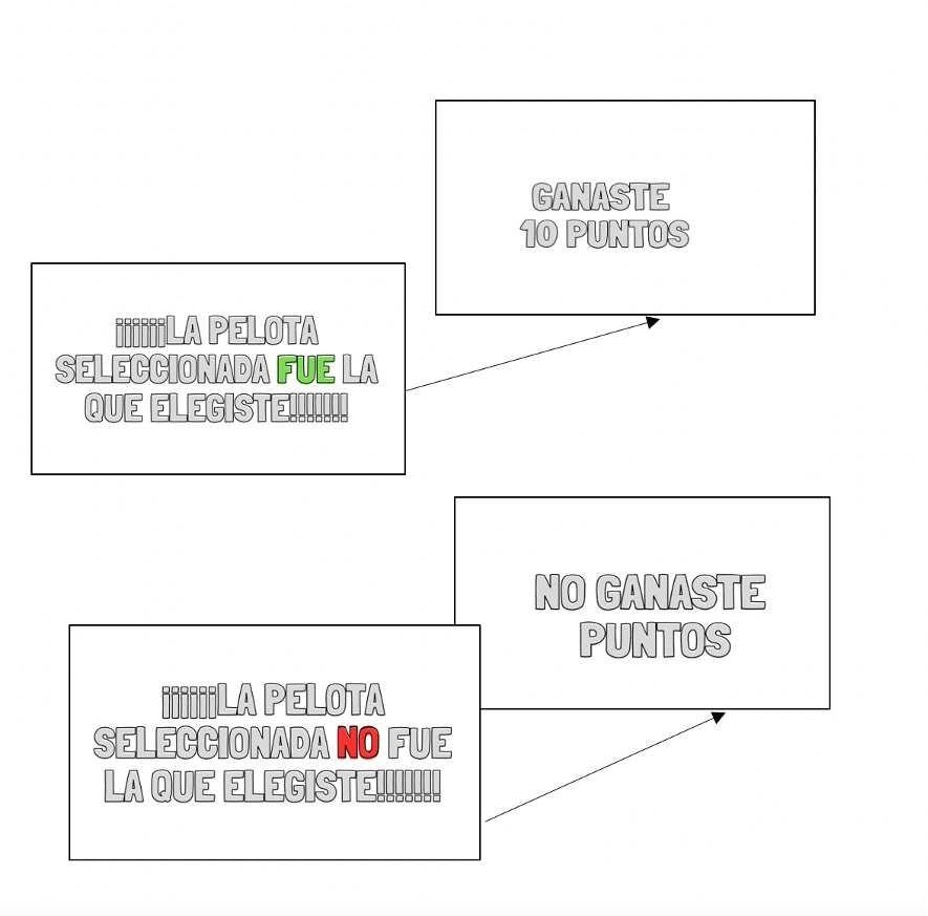

Supplement: S5 Fig — (TIF) [file pone.0290683.s005.tif]

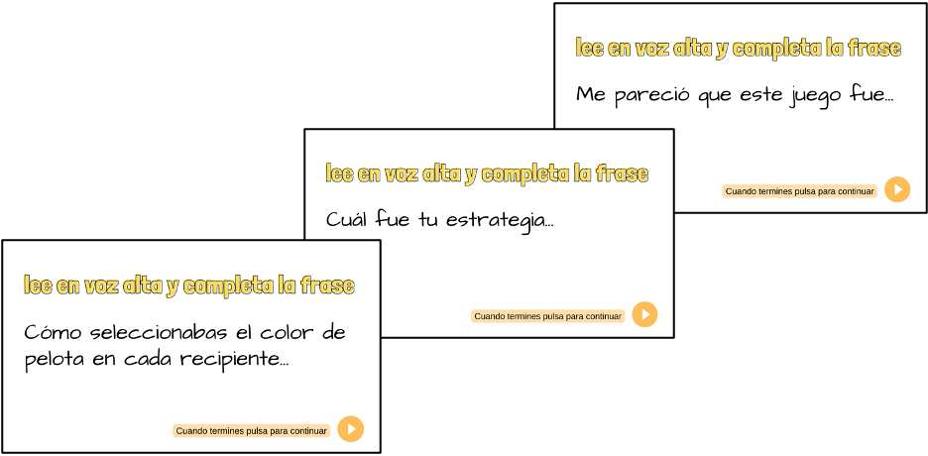

Supplement: S6 Fig — (TIF) [file pone.0290683.s006.tif]
